# Coping strategies of caregivers of persons with a disability attending a special education Center in Abakaliki, Southeast Nigeria: a cross-sectional study

**DOI:** 10.11604/pamj.2021.39.249.26884

**Published:** 2021-08-18

**Authors:** Chinonyelum Thecla Ezeonu, Dorathy Chinwe Obu, Olapeju Wunmi Daniyan, Uzoma Vivian Asiegbu, Oluchukwu Oyim-Elechi, Linda Obianuju Edafioghor, Kenneth Johnson Okoro

**Affiliations:** 1 Institute of Child Health, Alex Ekwueme Federal University Teaching Hospital, Abakaliki, Ebonyi State, Nigeria,; 2 Department of Pediatrics, Ebonyi State University, Abakaliki, Ebonyi State, Nigeria,; 3 Department of Pediatrics, Alex Ekwueme Federal University Teaching Hospital, Abakaliki, Ebonyi State, Nigeria,; 4 Department of Nutrition and Dietetics, Alex Ekwueme Federal University Teaching Hospital, Abakaliki, Ebonyi State, Nigeria

**Keywords:** Caregiver, child, coping, disability, Nigeria

## Abstract

**Introduction:**

coping strategies are specific efforts that individuals use to tolerate or minimize stressful events. Most caregivers´ of children with disabilities must adjust to their social life to cope with the responsibility of caring for a child with disabilities. This study was carried out to assess caregivers´ coping strategies in raising a child with a disability in a resource-poor country.

**Methods:**

a researcher-administered questionnaire adapted from the standard COPE (Committee on Publication Ethics) inventory was used on consenting respondents recruited from a designated special education school. Coping responses were graded on a five-point Likert scale and data were analyzed using computer software SPSS version 22.

**Results:**

the mean age of the respondents was 42.75 years. Males constituted 30% (12/40) whereas females constituted 70% (28/40). The diagnosed disorders amongst their children/wards were speech and hearing impairment (32.5%), cerebral palsy (12.5%), learning disability (10%), autism (10%), Down's syndrome (15%), epilepsy (20%). Most caregivers exhibited active coping (MNR 3-4) especially in areas of planning and seeking professional help. Turning to religion and acceptance of the situation (MNR 4-5) were common emotional coping strategies noted but most of these had no significant relationship with gender or income. Caregivers with lower earnings tend to pay more attention to the child´s disability than concentrating on other activities.

**Conclusion:**

the findings support that religious belief provides endurance and resistance to people dealing with stress while low socioeconomic status negatively affects the ability to focus on other activities during stress.

## Introduction

Disability is a general term that encompasses impairments, activity limitations, and participation restrictions experienced by an individual [1]. It can be physical, intellectual, sensory, psychiatric, neurological, and cognitive. Approximately 10% of a given population is probably disabled, and up to one in five of the world´s poorest have a disability [2]. In Nigeria, about 14 million people are living with disabilities [3]. Disability is both a cause and a consequence of poverty [4,5], reducing access to education, employment, opportunities, and resources [6]. It is documented that disability mostly affects vulnerable and marginalized people usually more prevalent among lower-income people in particular women, children, and older people [7]. Disability is a burden and a source of social stigma on the affected child and the caregivers. Disabled children also suffer from neglect, deprivation in society, social exclusion, and separation from other children. Such negative attitudes of isolation and rejection, potentially have a psychological and mental impact on the parents [8,9].

In most resource-poor settings, few organized systems accommodate the needs of the disabled. It more often takes a charity organization or a Non-governmental Organization or a religious group to set up a place for these disabled persons. All these leave the parents essentially to cope with the burden and responsibilities of caring for their disabled child using any possible strategy. Coping strategies are the specific efforts, both behavioral and psychological, that individuals use to tolerate, reduce, or minimize stressful events [10]. Coping with a child with a disability can be quite stressful and challenging to their caregivers, leading to adjustments in their social life [11]. Commonly used parental coping strategies include the use of instrumental social support, active coping positive reinterpretation, suppression of competing activities, and emotional support [12]. In this study, the authors sought to assess the coping mechanisms used by the caregivers of children with a disability attending a special education school, run by a religious organization in Abakaliki, the capital city of Ebonyi state. It is expected that our findings will give us some idea of the caregivers´ attitude towards disability and possibly proffer solutions to discourage negative attitudes. Primary objective: to determine caregivers´ coping strategy in raising a child with a disability in a resource-poor zone in South-East, Nigeria. Secondary objective: 1) to determine the distribution of disabilities among the study population. 2) to determine the association between socio-demographic characteristics and coping strategies used by the caregivers.

## Methods

**Study site and duration**: the study was conducted in June 2019 at a designated Special education school within the Abakaliki metropolis, Southeast, Nigeria. Abakaliki is the state capital of Ebonyi state. The main occupation of the people is subsistence farming, while some are traders, artisans, and public servants.

**Study population**: the study population consisted of caregivers of children diagnosed with a disability attending a special school located in the Abakaliki metropolis. Sixty caregivers had their children registered in the facility

**Study design**: the study design was cross-sectional.

**Eligibility criteria**: caregivers who had lived with a child with a physical or mental disability for at least two years or more and gave informed consent were included in the study while those that did not consent were excluded.

**Subject recruitment**: using the school´s directory which contains caregivers´ phone numbers and addresses, a total of sixty (60) invitations were sent out to caregivers by phone calls, letters, and text messages informing them about the research procedure and objectives. Forty-five (45) caregivers responded to the study, out of which five (5) opted out. Therefore, forty (40) caregivers participated in the study.

**Data collection method**: a total of 40 participants participated in the study. The study assessed the coping styles of caregivers of children with disabilities using researcher administered questionnaire adapted from the standard COPE inventory developed by Carver *et al*. [13]. This questionnaire was used to assess a broad range of coping responses by the caregivers. Responses were rated on a 5-point Likert scale, (1) never (2) rarely (3) sometimes (4) very often (5) always. Information on the socio-demographic profile of the caregivers was also obtained. The interview was carried out in the school office with one participant at a time to ensure and maintain privacy and confidentiality.

**Ethical approval and patient consent**: ethical approval was obtained from the Ebonyi State University Research Ethics Committee. The consent of the research subjects was obtained after due explanation of the method and purpose of the research. Confidentiality of information was assured by maintaining the anonymity of records and ensuring privacy during data collection.

**Data analysis**: the data collected were analyzed using Statistical Package for Social Sciences (IBM SPSS, version 22 Chicago, USA). Descriptive statistics (mean and standard deviation) were calculated for continuous variables while frequency and percentage were calculated for categorical variables. The mean score was calculated and rated. Chi-square was used to determine the association between dependent (coping strategies) and independent variables (socio-demographic profile). The level of statistical significance was achieved if p<0.05 at a 95% confidence level.

## Results

**Socio-demographic characteristics of respondents**: the age range of the respondents was 27-69 years with a mean age of 42.75 years. Males constituted 30% (12/40) whereas females constituted 70% (28/40) respondents. All were of Christianity religion with half of them of catholic denomination (50%), 10% Anglican while the rest were distributed across several other denominations. The age range of clients with a disability in the study area was between one year and 20 years. About eight of them (20%) were above the age of eighteen years but still needed to be cared for just as a child. There were 47.5% (19/40) males and 52.5% (21/40) females. Over 95% of the caregivers had an idea of the child´s diagnosis. The age at which diagnosis was made ranged from birth to 9 years of age, with 20% being at the age of 1 year, followed by the age of 3 years. Observed marital status of the caregivers before the diagnosis of the disability was obtained as 87.5% married, none widowed, 10% single, none separated. However, the status after the diagnosis showed a drop in the married group to 82.5%, 5% became widowed, 10% remained single, 5% had separated from their spouse. About 15% of the caregivers were poor, earning less than a dollar per day ($1 = N360) as at the time of the study), a greater percentage were of middle-income status (Table 1).

**Table 1:** total household income per month of the caregivers

Household income per month	n=40(percentage)
<$28	6(15)
$28-$83	8(20)
$83-$139	8(20)
$139-$194	8(20)
$194-$278	1(2.5)
$278-$417	2(5.0)
≥$417	7(17.5)
**Total**	**40(100)**

**Distribution of the various types of disabilities diagnosed**: speech and hearing impairment was the commonest diagnosis among these children needing special education while epilepsy was the least (Table 2).

**Table 2:** distribution of the various types of disabilities diagnosed

Diagnosis	n=40(percentage)
Speech and hearing impairment	13(32.5)
Cerebral palsy	5(12.5)
Learning disability	4(10.0)
Autism	4(10.0)
Down syndrome	6(15.0)
Epilepsy	8(20.0)
**Total**	40(100)

**Caregivers´ perceived causes of disability of their children/wards**: medical causes were reported in 35%, physical injury in 12.5%, and spiritual attacks in 17.5% of the caregivers whilst 32% did not have any idea about the cause of the child´s problem.

**Coping strategies used by the caregivers**: commonly utilized coping strategies by the respondents were turning to religion, acceptance, planning, and active coping whereas focusing on and venting emotions and alcohol-drug-social disengagement was the least utilized (Table 3).

**Table 3:** the mode and median frequency of coping strategies used by the caregivers

Coping strategy	Mode	Median	MNR
**Active coping**			
I take additional action to try to get rid of the problem	3.00	3.00	3.40
I take things slowly as they come	3.00	3.00	3.95
**Planning**			
I try to think of a better way to tackle the problem	5.00	4.00	3.95
**Suppression of competing activities**			
I pay attention more to this problem than concentrating on the other activities	3.00	3.00	3.13
**Restraint coping**			
I restrain myself not to act too quickly	3.00	3.00	3.30
**Seeking social support for instrumental reasons**			
I try to get advice from people who have a similar experience	3.00	3.00	2.95
I try to get professional help	5.00	4.00	3.90
**Seeking social support for emotional stress**			
I discuss the way I feel with friends/relatives	3.00	3.00	3.00
**Positive reinterpretation and growth**			
The experience has made me a changed person	4.00	4.00	3.53
I tried to see a positive thing in this situation	5.00	4.00	3.95
**Acceptance**			
I learn to live with the situation	5.00	4.00	3.95
**Turning to religion**			
I put my trust in God, hoping for the best	5.00	5.00	4.68
I pray more than I used to	5.00	5.00	4.22
**Focus on and venting of emotions**			
I get angry with people around me	1.00	1.50	1.95
**Behavioral disengagement**			
I tell myself I can´t handle it, so I stop trying	1.00	1.00	1.88
I put much effort into solving the problem	5.00	4.00	4.03
**Denial**			
I refuse to believe that it has happened	3.00	3.00	2.75
**Mental disengagement**			
I turned to work or another activity to take my mind off things	1.00	3.00	2.58
I slept more than usual	1.00	1.00	1.65
**Humor**			
I laugh about the situation	1.00	2.00	2.00
I make jokes about the situation	1.00	1.00	1.53
**Alcohol-drug-social disengagement**			
I try to make myself feel better by taking alcohol	1.00	1,00	1.18
I try to make myself feel better by taking smoking	1.00	1.00	1.08
I try to make myself feel better by taking drugs or medication	1.00	1.00	1.05

**Association between socio-demographic characteristics and coping strategies used by the caregivers**: most of the coping strategies had no significant relationship with gender or income. However, on the habit of suppression of competing activities, it was found that amongst those with household income ranging from $28-$83, that they paid more attention to the existing problem than concentrating on other things. Whereas the higher income earners from $417 and above, never, or rarely paid attention to the problem at the expense of other activities. This was statistically significant (p=0.002). For social support, this varied in degrees with gender. Most women would try to seek social support than males (p=0.005). Active coping strategies such as taking additional actions to get rid of the problem or seeking advice from people did not have any significant relationship with the type of disability. Positive reinterpretation of the problem was significantly associated (p=0.03) with the type of disability, being more for speech/hearing impairment and neutral for Down´s syndrome. Turning to God and hoping for the best was significantly related to the forms of disabilities (p=0.004).

## Discussion

This study was carried out to determine the various ways caregivers cope with their child´s disability since individuals and families respond to stress in various ways. There was an observed slight drop in the marital status of the caregiver when the diagnosis of their children was made known to them. This finding suggests that knowing that a child has a disability may lead to family separation. Cauda-Laufer [14] and Witt *et al*. [15] found a higher divorce rate in parents of children with disabilities compared to parents of children without disabilities. Vulnerability to divorce can be explained by a high level of parenting demands and stress of raising a child with a disability leading to a reduction in receptiveness to the needs of one´s spouse during these times [14]. Their work lives, social lives, and emotional states are also affected [15]. This emphasizes the need for an effective support system that may go beyond the immediate family system to include respite care as these will give the affected caregivers respite enabling them to have quality time to engage in other activities, thus reducing their stress [16]. Also, they will spend quality time together and are relieved temporarily of that magnitude of care.

The predominant disability seen in this school of special needs was speech and hearing impairment. The reason may be because most members of society tend to have a better understanding of speech and hearing impairment amongst other disabilities in children [17]. Also, burnout syndrome on the part of caregivers may make them withdraw already registered children with severe disabilities in the school of special education. This may account for the varying distributions of diagnosed impairment of children attending this school and not a true reflection of what exists in society. The belief that the mentally retarded are not educable may contribute to the low registration in special need schools. Hence public enlightenment campaigns and useful support systems need to be instituted to address this to improve school attendance of these affected children. Similarly, Ologe and Akande [18] reported deafness as the commonest disability in the handicapped school in Ilorin while Ganjiwale *et al*. [19] in India reported speech impairment. Contrary to this finding, some researchers have reported attention deficit hyperactivity disorders as the commonest developmental disabilities in their studies [14,20].

More females were registered in this school of special needs. Although culturally Africans are believed to value male children more than females, this finding may be a result of the small study population or because those who are enrolled may be the ones whose caregivers can afford to keep in the school. Similarly, Alkhaledi [20] in Saudi Arabia reported female preponderance. Other studies reported male preponderance [18,19,21]. A significant number of the caregivers perceived that the cause of their child´s disability was medical, while quite a few of them did not know what caused their child´s disability. Knowledge of the cause of a disability is likely to help the caregivers cope better in caring for affected children and also prevent such recurrence where possible [22]. This is comparable to findings by other researchers [14,18,20]. Effective use of coping strategies has been reported to help caregivers adapt well to the stress of caring for a disabled child [21]. In this study, turning to religion, planning, acceptance, positive reinterpretation, active coping, and seeking social support were the commonest coping strategies used by the caregivers of children with disabilities. These strategies grouped under problem-focused coping strategy appear to be the most preferred coping strategies since they have a positive influence on reducing the stress associated with raising a child with a disability [23]. Active emotional coping strategy comprising of restraint coping, acceptance, positive reinterpretation and growth, seeking social support for emotional stress, focus, and venting of emotions were the next utilized coping strategies in this study. Family cohesion and cooperation have been reported as factors most helpful in coping [24]. Ntoumanis *et al*. [25] had suggested that the use of emotional-focused coping mechanisms may lead caregivers to reappraise stressors previously thought to be stress resulting in effective coping to deal with the situation.

The favored use of both groups of strategies by most caregivers in this study may be because caregivers felt that a realistic outlook of child´s disability, acceptance of the situation, seeking for professional solution, religious belief that there is a reason for such happening and most importantly leaving everything to faith may help them handle the situation better. The soothing effect of religion may not be overlooked in coping. Religious belief provides endurance and resistance to people while they are coping with stress and stressful situations [26]. This type of strategy may be a result of appropriate counseling provided either at the point of the diagnosis in the hospital, at the special school by the teachers, or even family friends during home visits. This highlights the need for thorough counseling of caregivers of children with such disabilities emphasizing the cause of the disability, natural history, and prognosis so that caregivers are well informed about their expectations of such children, therefore adapting well in providing needed care for such children. Ganjiwale *et al*. [19] observed the predominant use of problem-focused solving and active emotional coping strategies with the emotional coping strategy being the most preferred in their study. On the contrary, Alkhaledi [20] reported poor scores towards the nine coping techniques studied among caregivers of children with physical special needs. These differing findings may be due to the cultural and religious differences of the respondents and the type of disabilities of affected children.

Seeking social support, behavioral disengagement, denial, focus on and venting of emotions, and alcohol-drug-social disengagement which is part of the avoidant emotional coping strategy was the least coping strategies used by the respondents in this study. Caregivers that made use of this strategy believed that not acknowledging and thinking about the problem would help them adapt to the child´s disability as such an extra burden is lifted off their shoulders. They discuss a child´s problem, resort to other ways of forgetting these problems without searching for a solution. They may face more stress later in life thus abandoning the care of the child. Complete education and information about the child´s disability if given to caregivers helps to reduce stress and also leads to the use of positive coping strategies among caregivers [27]. Household income was associated with the suppression of competing activities. Caregivers with higher earnings tend to focus their attention on other things than low-income earners. The reason may be because the former may have jobs/businesses that keep their mind preoccupied. Also, caregivers with higher income are more likely to afford adaptive equipment and other support needed for the index child compared to low-income earners. Thus, reducing the stress associated with taking care of a child with a disability. Most women would try to seek social support than men, the reason may be because women constituted a majority of the study population of the caregivers and also women´s inclination to seek social support [28]. Ammari *et al*. [29] reported that parents of children with special needs tend to seek social support from social media platforms like Facebook groups even though the expected support needed may not be achieved.

## Conclusion

This study has highlighted the coping mechanism mostly used by caregivers which includes turning to religion, planning, acceptance, and positive reinterpretation, active coping, and seeking social support. Caregivers with lower earnings tend to pay more attention to the child´s disability than concentrating on other activities. Therefore, there is a need for financial and social support for caregivers of children with disabilities so that they can cope better with the stress and challenges associated with caring for these children. Since religious belief provides endurance for individuals for coping with a stressful situation, it may not be out of place to encourage the psychosocial benefits of religion and counseling of caregivers as a motivation for a more positive outlook on life and its potential stress.

### What is known about this topic

- Raising a child with a disability is fraught with physical, mental, and emotional stress which can overwhelm caregivers of children with disabilities;
- There are limited societal supports available for caregivers of children with disabilities;
- Various strategies are used passively or actively by individuals to cope with the challenges of raising a child with a disability. However, the ways parents interpret, perceive, and manage the stress involved in caring for a disabled child differ especially in a resource-poor setting like Abakaliki.

### What this study adds

- Speech and hearing impairment were the commonest disabilities observed in the study area, highlighting the need for rehabilitative activities targeted at this group;
- Active problem-focused and active emotional coping strategies such as seeking solutions from professionals, turning to religion, planning, acceptance, and positive reinterpretation of the situation were commonly utilized by caregivers in this study irrespective of gender, age, or social class;
- Avoidance coping strategies such as denial or alcohol-drug-social disengagement were the least coping strategies used by the respondents which should be encouraged.
